# How Exceptional Is the Ear?

**DOI:** 10.1007/s10162-025-00988-z

**Published:** 2025-05-12

**Authors:** Christopher Bergevin, Dennis M. Freeman, Allison Coffin

**Affiliations:** 1 Department of Physics & Astronomy, York University, Toronto, ON Canada; 2 Department of Electrical Engineering & Computer Science, Massachusetts Institute of Technology, Cambridge, MA USA; 3 Department of Integrative Physiology and Neuroscience, Washington State University, Pullman, WA USA; 4 Bellucci Translational Hearing Center and Department of Biomedical Sciences, Creighton University, Omaha, NE USA

**Keywords:** Active hearing, Auditory biomechanics, Biophysics, Cochlear mechanics, Otoacoustics

## Abstract

Studies of hearing often conclude that the ear is “remarkable” or that its performance is “exceptional.” Some common examples include the following: $\triangleright$ the ears of mammals are encased in the hardest bone in the body; $\triangleright$ the ear contains the most vascularized tissue in body; $\triangleright$ the ear has the highest resting potential in the body; $\triangleright$ ears have a unique “fingerprint”; $\triangleright$ the ear can detect signals below the thermal noise floor; and $\triangleright$ the ear is highly nonlinear (or highly linear, depending upon who you ask). Some claims hold up to further scrutiny, while others do not. Additionally, several claims hold for animals in one taxon, while others are shared across taxa. Most frequently, our sense of wonder results from the differences between ears as products of natural selection (over eons) and artificial systems as products of engineering design. Our goal in analyzing claims of remarkable or exceptional performance is to deepen our appreciation of these differences.

Introduction Given the ubiquity of sound in everyday life, there are myriad connections between hearing research and the general interests of non-scientists. From listening to birds singing in the morning, attending a concert at a music hall, or engaging in casual conversation with friends, hearing provides a means of communication and appreciation of the world around us. As scientists and clinicians, we can form a more nuanced appreciation of auditory science, and one approach is to consider the “remarkable” [1, 2] functionality of the mammalian cochlea as a biological detector of sound. That is, the cochlea (i.e., the auditory component of the inner ear) exhibits numerous characteristics that from a biophysical point of view are impressive, given limiting constraints such as thermal noise, the operating range of neural responses, and fluid mechanics.

To illustrate, hearing intrinsically spans a broad range of spatial dimensions. The length scale (in meters) of the following hearing-related structures and phenomena spreads across 16 orders of magnitude:

- $\diamond$ $10^{-12}$ m = displacement of the eardrum in response to sound at the threshold of hearing [3]
- $\diamond$ $10^{-9}$   = thermal noise agitation of hair cell bundles [4]
- $\diamond$ $10^{-8}$    = step size of a myosin motor
- $\diamond$ $10^{-7}$    = displacement of the eardrum in response to damage-inducing sounds
- $\diamond$ $10^{-5}$    = width of a hair cell
- $\diamond$ $10^{-2}$    = length of the cochlea
- $\diamond$ $10^{0}$       =   “our” size (human height)
- $\diamond$ $10^{-2}$ to $10^{4}$ = the acoustic world we commonly perceive

Taken at face value, this span does appear remarkable! However, it’s not clear how the range of 16 orders of magnitude is interesting since the dimensions listed above are not directly comparable to each other. For example, it is not clear how to meaningfully compare the displacement of the eardrum in response to damage-inducing sounds to the width of a hair cell. By contrast, the displacement of the eardrum in response to damage-inducing sounds can be meaningfully compared to the displacement of the eardrum at the threshold of hearing, since their ratio defines dynamic range, which is a useful engineering metric.

We are especially interested in characterizing properties of the ear that are “remarkable” in comparison to limits set by physics and engineering. For example, one popular review with well over one thousand citations [6] stated “The internal ear is an evolutionary triumph of miniaturization.” With eons of selective pressures, ears have evolved in myriad forms that push the limits of what is physically possible [7]. In this manuscript, we consider claims about “remarkable” properties of ears that have resulted, including a perspective that functional optimizations within the ear may potentially conflict (e.g., sharp frequency selectivity versus high temporal acuity versus “miniaturization”) [8]. Our goal is to illustrate some of the fascinating products of natural selection and to better understand how their behaviors can be surprising based on our engineering experiences and the physical sciences.

## Claims of Remarkableness

Numerous assertions regarding the ear are commonly mentioned — in research papers, formal presentations, and even arguments with colleagues at conference happy hours. To set the stage for our review of the remarkableness of the ear, here we state several claims, without reference, justification, or assertion. It is important to note that in this list, “the ear” refers to anatomical structures of the outer, middle, and inner ear. We break down the specific frame of reference later in the paper as we review each claim.

(A) The displacement of the eardrum at the threshold of hearing is $10^{-12}$ m.
(B) The ear is encased in the hardest bone in the body.
(C) The ear contains the smallest bones in the body.
(D) The ear contains the most vascularized tissue in the body.
(E) The ear has the highest resting potential in the body.
(F) The ear is the fastest (mechanical) part of the body.
(G) The ear can detect signals below the thermal noise floor.
(H) The ear accommodates an enormous dynamic range in terms of incident sound energy.
(I) The input range of the central nervous system  is much smaller than that of audible sound levels.
(J) The ear not only detects sound but emits it as well.
(K) The ear has a built-in amplifier.
(L) The ear has a unique “fingerprint.”
(M) Cochlear waves play a crucial role in hearing.
(N) The ear is poised on the verge of an instability.
(O) The ear is highly nonlinear.
(P) The ear is very fragile.
(Q) Human hearing exhibits relatively exceptional tuning sharpness.

These claims range from provocative (A)–(G), to established orthodoxy in the field (H)–(L), to hotly debated (M)–(Q). In many cases, these claims are not simply true or false, and the reality lies somewhere in between. Further, direct evaluation of their validity is difficult in part due to the wide variety of animal models employed in auditory physiology. For example, how does one best compare in vivo motions of the mouse basilar membrane to in vitro bullfrog vestibular hair cells decoupled from the otoconial mass? Nonetheless, examining the rationale for these claims serves to help illuminate many of the current (known and unknown) unknowns in auditory science. The goal of this review is to provide a broad review of scientific literature relevant to the basis for these claims. Is the ear indeed remarkable? We urge the reader to consider our arguments and decide for themselves.

## Justifications and/or Refutations

### $\rhd$ (A) The displacement of the eardrum at the threshold of hearing is $10^{-12}$ m

**Fig. 1:**
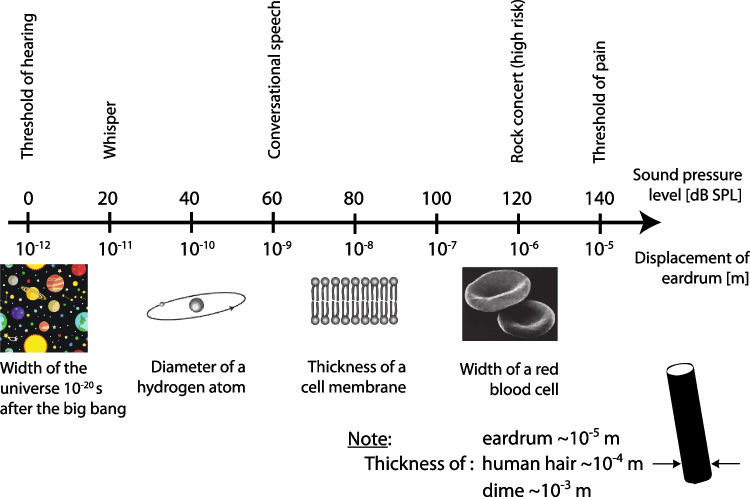
Schematic to show the comparison between (top) sound pressure level at the eardrum and representative sounds at those levels and (bottom) the corresponding displacement of the eardrum [3]. Note that the human eardrum itself is very thin: only about 0.05–0.12 mm thick [5]. For the bottom plot, several objects with representative dimensions are also shown for comparison, as is the thickness of a human hair

**True** — Laser Doppler vibrometry (LDV) is a common methodology for auditory-related measures capable of detecting displacements that are much smaller than the wavelength of visible light. Measurements suggest that, at threshold, the human eardrum (specifically, the tympanic membrane, TyM) moves on the order of one picometer (pm; i.e., $10^{-12}$ m) [3]^1^. By comparison, the diameter of a hydrogen atom is 100 pm — two orders of magnitude larger! Fig. 1 provides some context for the remarkably small magnitude of this TyM motion and connects to Claim (H) regarding the enormous dynamic range of the ear^2^.

We note that the eardrum is not a piston, in that it does not exhibit uniform motion across its surface. In fact, the sound-evoked motion of the eardrum can be highly complex (e.g., numerous higher order modes) and is not completely understood [9, 10]. Nonetheless, we provide here a simple back-of-the-envelope calculation that suggests a 1 pm displacement at threshold physically makes sense [11]. A commonly cited value is that at our most sensitive frequencies, about 1–4 kHz [12], the sound pressure threshold level ($p_o$) at the eardrum is $2 \times 10^{-5}$ N/m$^2$. This value sets the decibel “SPL” (sound pressure level) reference at 0 dB SPL. The corresponding threshold intensity ($I_o$) is defined at $p_o^2/z_o$, where $z_o$ is the characteristic acoustic impedance and is about 400 Pa s/m for ambient air. Thus, $I_o \approx 10^{-12}$ W/m$^2$. For sinusoidal oscillations, the air pressure depends upon several aspects of the gas itself as follows

1 $$\begin{aligned} p = \frac{2\pi B A}{\lambda } \end{aligned}$$

where *B* is the bulk modulus of the gas, $\lambda$ is the wavelength (i.e., wave speed/frequency), and *A* is the amplitude of oscillation of the gas molecules. Rearranging and solving for *A*, one finds a value of approximately $10^{-11}$ m, roughly in the same ballpark as the LDV measurements [3]. This means that the eardrum is moving a similar amount as the air itself in response to the acoustic pressure, which may indicate a close match in impedance between air and the TyM that reduces energy reflection. Further, we can estimate the amount of energy incident at the ear at the threshold. The human eardrum has roughly an area of 0.5 cm$^2$, or $5 \times 10^{-5}$ m$^2$. Given the intensity value stated above, this would correspond to $5 \times 10^{-17}$ W. Assume the ear requires 0.2 s to integrate a sound over near threshold for detection. The total incident energy would then be $10^{-17}$ J (approximately $2000\,k_BT$), which has been shown [13] to be comparable to the incident photon energy required for visual detection!

The above calculations are specific to the human ear and different animal species may exhibit different threshold displacements. Consider the gerbil for example, where it was reported that the umbo (where the tip of the manubrium of the malleus attaches to the TyM) exhibited an approximately 1 nm amplitude for a 10 kHz stimulus at 80 dB SPL [14]. Auditory nerve thresholds at 10 kHz for gerbil are about -5 dB SPL [15]. Assuming linearity and extrapolating downwards [3, 16], that would lead to an umbo displacement of 0.1 pm, about an order of magnitude smaller than the values noted above for humans.^3^ The reason for this discrepancy is not clear but may stem from the spurious assumption about linearity (e.g., eardrum motion does not scale in direct proportion with incident sound amplitude at the lowest levels, perhaps due to amplification; see Claim (J)). As a non-mammalian example, Manley [17] reported that for the Tokay gecko, eardrum displacements were 1 $\mu$m at 1 kHz for 100 dB re 2.0$\times 10^{-4}$ dynes/cm$^2$, equivalent to 100 dB SPL. This would translate (again, scaling linearly) to a 10 pm displacement at 0 dB SPL. Based on these calculations, and perhaps unsurprisingly, evolution has yielded remarkable ears across the animal kingdom.

### $\rhd$ (B) The ear is encased in the hardest bone in the body

**Fig. 2:**
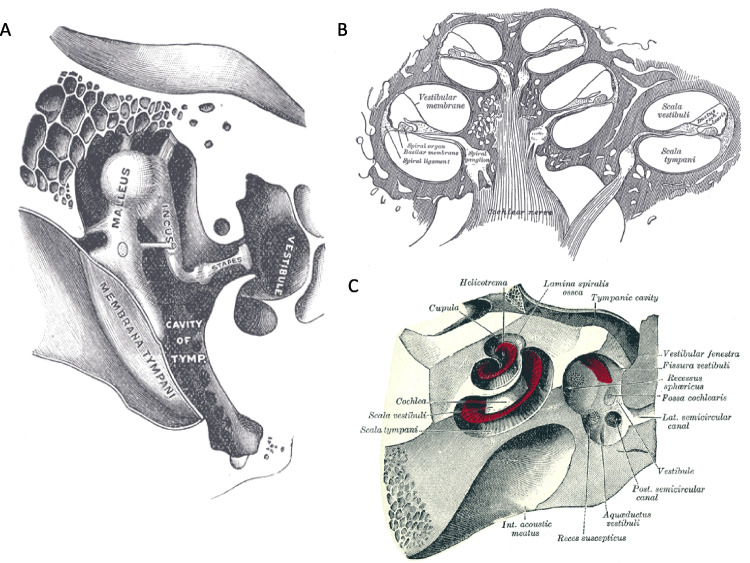
**A** Cross-section through the base of the human skull, showing the middle ear space heading towards the cochlea. Note that around the cochlea, the bone appears denser with fewer cavities relative to the nearby mastoid area. Cross-sections of the cochlea (**B**) and temporal bone (**C**) are also shown, visually indicating the relatively dense bone surrounding the inner ear. All images are from [26] and obtained from the Wikimedia Commons where they are in the public domain

**Partially True** — The inner ear is partly encased in the petrous part of the temporal bone, also referred to as the otic capsule (Fig. 2). The name stems from the Latin word *petrosus*, which translates to rocky, stone-like, or petrified [18]. One suggestion is that this name derives from the notion that one primary purpose of the bone is for “offering protection from impacts, falls, and other trauma” [19], a helpful feature for the seemingly fragile inner ear. Another viewpoint is that the denser the bone, the higher the bulk modulus that would thereby affect acoustic propagation via bone conduction.^4^

The density of the temporal bone is non-uniform. While the petrous portion, which sits on the floor of the cranial cavity, appears relatively dense in areas around the bony labyrinth, the mastoid portion has sections that are comparably sparse (e.g., holes to allow blood vessel passage) [24]. From a clinical perspective, this variation in density is significant, as it greatly improves surgical approaches (i.e., easier bone drilling) for procedures such as a mastoidectomy, a common approach for implantation of cochlear implants. In mammalian species used for auditory research such as gerbil, the cochlea bulges out from the base of the skull into the air-filled middle ear space (bulla). Thus, much of the murid cochlea only has a thin layer of bone around it, which may have functional consequences relative to other mammals where the cochlea is deeply embedded (e.g., see [25] for a discussion of marine mammals).

There are anecdotal accounts suggesting that temporal bone density may in fact be the highest in the body. These stem from temporal bones being used for extraction of DNA in archaeological studies (e.g., [27]). Consider this quote from [28]: “The only skeletal element for which a systematically higher endogenous DNA content compared to other skeletal elements has been shown is the petrous part of the temporal bone.” These reports allude to the petrous part of the temporal bone as being the “densest” in the body, pointing towards a 1999 tomography study [29] that claimed “The most dense element examined for each species is the petrous temporal, which is approximately 10 percent denser than the middle shaft portions of bones.” That study, however, did not examine human bones, but rather “bovids, cervids, and equids.”

Common sense dictates that other (non-bone) structures are likely the “hardest” body structures. For example, teeth are used in forensic dentistry to identify persons whose bodies have otherwise been destroyed in a fire (e.g., [30]). Another study [29] cited mineral density values of approximately 1.50 and 1.05 g/cm$^3$ for enamel and dentine portions, respectively, while calculating cortical bone density in the range of 0.95–1.15 g/cm$^3$. It is unclear whether temporal bones fall into the upper end of this density range. Regardless of the final calculations, temporal bones are hard, and partially encapsulate the inner ear.

### $\rhd$ (C) The ear contains the smallest bones in the body

**Likely True** — Human anatomy textbooks commonly refer to the three ossicles (incus, malleus, and stapes) as being relatively small, even the “smallest” [24] or “tiniest” [31] in the body. Research articles also make similar assertions (e.g., [32, 33]), as do books on auditory sensation (e.g., [34]). Even the Guinness World Records lists the stapes as the winner in the “smallest bone” category. Relatedly, the stapedius is also commonly pointed to as the smallest striated and skeletal muscle in the body. But what is the rationale for such claims, as references are rarely, if ever, provided?

There are 200 bones in the human body [35], although this number changes with age as some bones fuse following birth. Unlike some bones in the body (e.g., skull), human ossicles are fully formed and ossified at birth, leading some to call the stapes the most “adult” bone in the developing human embryo [36, 37]. Of the three ossicles, the stapes have the shortest maximum length.^5^ The footplate (which makes the final ossicular connection to the inner ear) is roughly elliptical and makes up the bulk of the stapes mass [41]. Roughly, typical dimensions are: footplate major and minor axes are 2.5 by 1.4 mm respectively (yielding a footplate area of approximately 3.5 mm$^2$), a height of 2.9 mm (i.e., distance from footplate to top of superstructure arch), and of a mass of 2.2–4 mg ([42], citing [43, 44]). These mass values support the argument that the stapes is the smallest bone in the human body.^6^

While the claim of “smallest” appears applicable to the human middle ear ossicles, there is considerable diversity in ossicles across taxa, such that this claim may not hold true in other species. For example, terrestrial non-mammalian vertebrates such as birds and reptiles only have a single ossicle, making it unclear whether the ossicle would still be the smallest bone in their bodies. Further, in some terrestrial mammals like elephants, the ossicles appear rather large and are thought to improve ground vibration detection (e.g., [45, 46]). Similar considerations may also apply to marine mammals [45]. Numerous allometric studies of the middle and inner ears exist (e.g., [47, 48]), and it will likely prove instructive in the future to connect comparative studies with functional considerations [49].^7^

To conclude this section, we quote the final paragraph from [36]: “*The stapes, then, is unique in its developmental history; it is the only annulet in the human body, and the most exquisitely fashioned bone in the entire supportive system. Beginning as an obscure cog in the respiratory machine, it becomes an almost indispensable part of a sensory apparatus (...) it need not be a giant to be important. Delicacy and mobility are required for the discharge of its physiological duties; to that end, the stapes has thrown away half of its osseous self and its very marrow. It is the anatomical patriot par excellence*.” As a point of contrast, a surgeon performing a stapedectomy might find this sentiment sophomoric, although a stapedectomized elephant might be very unhappy!

### $\rhd$ (D) The ear contains the most vascularized tissue in the body

**Unlikely** — First, how is the degree of vascularization in the ear quantified? After all, the heart and lungs, and even tumors [53], are highly vascularized tissues. From an energy perspective, metabolic requirements for mechanical sensing systems are complex, and the vascular system is essential in this regard (see the Secomb & Pries chapter in [54]). For the cochlea, the intuitive idea is that energy is required to generate the large resting potential in the scala media (SM), [55], as explored in Claims (E) and (K). Through that lens, a high degree of vascularization makes sense: Relatively high blood oxygenation would help sustain the underlying metabolic processes needed to overcome the large electro-chemical gradients, and thereby fuel cochlear amplification.

There are two different principal pathways that supply blood to the inner ear [56]. The first pathway comes from the carotid artery and innervates the otic capsule, which constitutes the bony walls of the inner ear and contains perilymphatic fluid. This bony labyrinth is composed of several vessels extending from the end branch of the external carotid artery (maxillary, auricular, and meningeal) [57]. The second pathway comes from the cerebellar pathway (part of the vertebral arteries) and innervates the “membranous labyrinth,” which contains endolymphatic fluid and is where the hair cells reside. More specifically, this branch is the labyrinthine artery [58], which is part of vertebrobasilar branch off the cerebellar artery. It runs in parallel with the vestibulocochlear nerve along the internal auditory meatus. The stria vascularis is a thin ribbon of tissue that runs along the lateral wall of the cochlea from base to apex and is innervated by this path [56, 59]. The stria has been noted as having a high concentration of blood vessels [12, 60], but such has not been quantified.

Let us now make a simple argument based upon the relative dimensions of the cochlea. An adult has approximately 5 L of blood, and at any given time: $\approx$ 7–10% in the heart, $\approx$ 9–12% in the lungs, $\approx$ 60–64% in the venous, and the remainder in the arteries and capillaries [24, 61]. While there is individual variation in cochlear dimensions [62], we can make several approximations. To first order, the volume of the cochlea is approximately 100 mm$^3$ ($\sim$0.0001 L). Presumably, only a small fraction (say 5%) would comprise vasculature, which is confined to the external wall and the spiral lamina. Thus, at any given instant, a comparably small fraction of an individual’s blood (say 0.000005%) is in a given ear, an estimate roughly consistent with [63]. Understandably, there is relatively little blood flowing to the ear compared to other parts of the body. However, this does not necessarily refute the claim that the ear contains the most vascularized tissue in the body in light of other dimensional considerations.

**Fig. 3:**
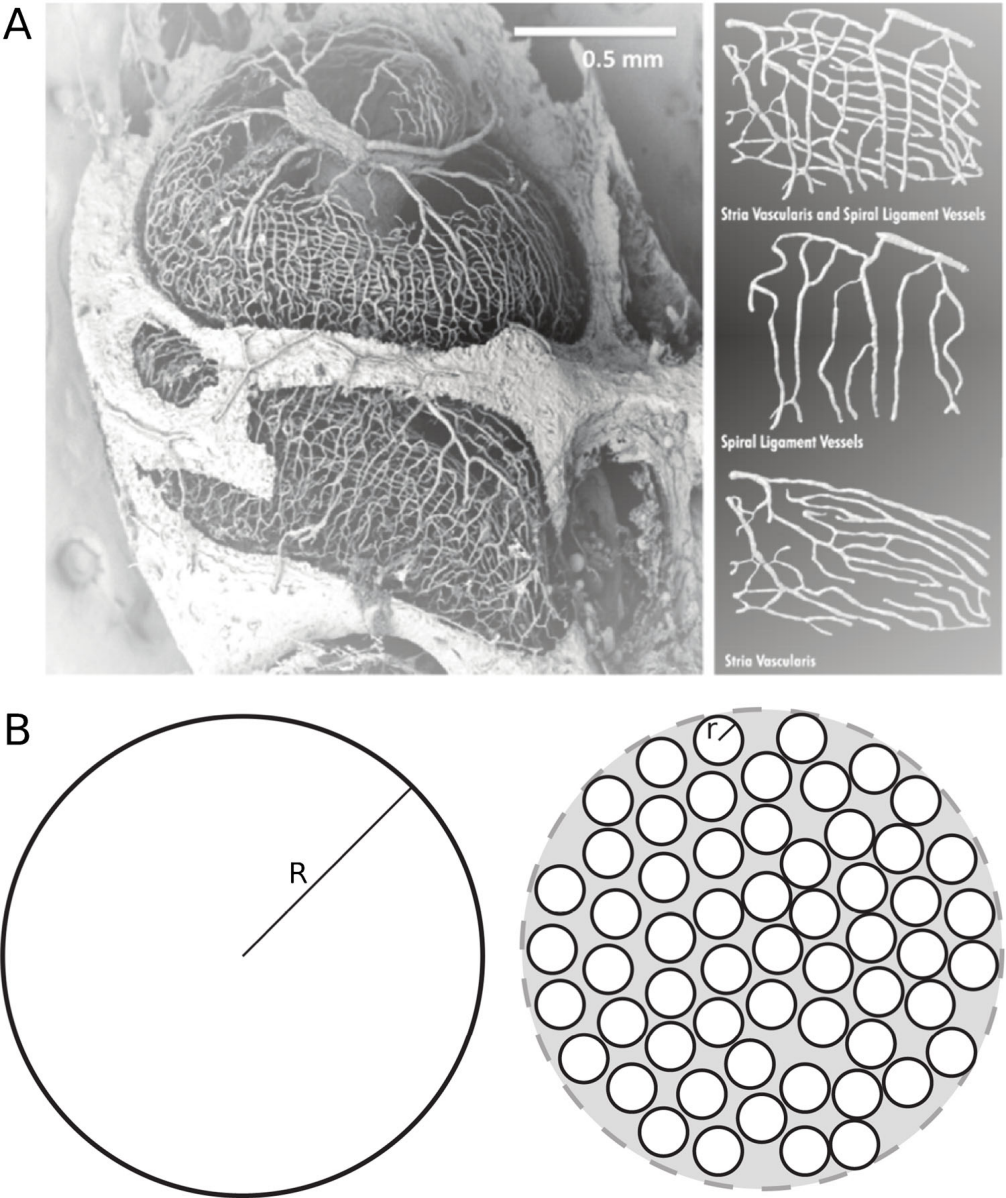
**A** Cochlear vasculature of the mouse via corrosion casting [64] (image used by permission of Springer Nature). Note that in the right panel, the top plot is a superposition of the two below (the bottommost showing the stria vascularis). **B** Heuristic schematic to consider the high degree of vascular branching. The left panel indicates a cross-section through a circular vessel of radius *R*. The right panel shows *N* (=58) vessels with radius *r*, where $10 r = R$. Even though the total cross-sectional area on the right is less (i.e, the ratio of the white area to the grey area is 0.58), the epithelial surface (i.e., total circumferential length of black lines) is much larger. Thus if epithelial surface (i.e., where transport occurs) is the key quantitative measure, the case on the right is “more vascularized” by nearly a factor of 6

Recent studies of mice using cast corrosion and electron microscopy have provided detailed microanatomy of the cochlear vasculature [64, 65]. From these data, it becomes apparent that there is a high degree of vascular branching within the stria vascularis (see Fig. 3A). One takeaway from these images is that while the cross-sectional area (in a plane slicing orthogonal through the stria) may be somewhat small, the relative epithelial surface for transport is large (see Fig. 3B). Seen this way, the large degree of branching supports the cochlea as being highly “vascularized,” despite a relatively small blood volume and a rather modest number of cells being fed to (i.e., only about 10000 hair cells per cochlea). At present, we do not know if the cochlear vasculature bifurcates comparatively more than other tissues. Therefore, we conclude that the cochlea is highly vascularized, but not necessarily the most vascularized tissue.

### $\rhd$ (E) The ear has the highest resting potential in the body

**Likely True** — First, let us confine considerations to membrane potentials only. That is, electrical potentials are generated across the membrane of a single cell (in contrast to cellular potentials that are generated by multiple cells). Second, we will only consider resting potentials and not activity-driven changes, such as action potentials.^8^ Assuming ionic concentrations and membrane conductances are in steady-state (e.g., homeostatic conditions), this potential is nearly constant. The size of this potential varies for different cell types and ionic conditions, but is typically negative, meaning the inside of a cell is negatively charged compared to the outside (in the range of 0 to −100 mV).

For the cochlea, hair cells have a relatively normal intracellular potential relative to electrical ground ($\approx -60$ mV). However, the stria vascularis actively pumps potassium ions (K+) against its electrodiffusive gradient. As a result, the endocochlear potential (EP) is created: a $+80$ mV potential in the endolymph of the scala media (SM) relative to the perilymph comprising scala vestibuli and scala tympani. Thus, hair cells experience a much larger transmembrane resting potential of $\approx 130-170$ mV across their apical surface relative to SM [66–69]. This cellular battery effectively helps drive mechano-electro transduction (MET) currents (i.e., hair bundle deflections causing K+ and calcium flow) and powers active processes. However, is this 130–170 mV the highest potential in the body?

There are numerous large trans-membrane potentials throughout the body in electrically excitable cells, such as neural action potentials (upwards of 140 mV; [70]) and cardiac cells. But as alluded to earlier, these are transient de–/re–polarizations, unlike the EP, which is relatively constant. A key facet to this large transmembrane resting potential is the unique nature of the scala media environment. To illustrate, the endolymph is considered unique in that it is an extracellular fluid that more closely resembles an intracellular fluid, in part by having a high potassium concentration [12].^9^ As a result, the scala media is at $+80$ mV relative to scala vestibuli, which leads to a $\approx 150$ mV difference between endolymph and the inside of the cell. The unique cochlear fluid partitions do indeed appear to generate the largest resting membrane potential in the body.

### $\rhd$ (F) The ear is the fastest part of the body

**True** — Time and spatial scales confound this statement. “Fast” is a relative term, as is “part of the body.” As a starting point, let us first consider the ear in comparison to other sensory systems. The “speed of smell” can indeed be considered fast, occurring on timescales of 10–100 ms [71, 72], though it is relatively slow compared to the ear’s response to oscillatory stimuli with periods of 0.01–10 ms. With regard to vision, the eye can be considered both fast and slow: Rhodopsin isomerization in visual phototransduction is extremely fast (i.e., femtosecond) [73], although one can readily hear a 6 kHz tone but not discern flickering in a light source at 60 Hz (see also [74]), two orders of magnitude slower. A key distinction is that signal processing in the cochlea is done hydromechanically — not neurally. This can be understood at least in part because the underlying components, primarily hair cell MET channels and motor proteins of the outer hair cell that produce motion via force generation, are much faster than the corresponding neural components (sodium and potassium channels). We first consider more generally how other parts of the body produce force, and then argue why the ear is biomechanically the fastest.

Generally speaking, individual molecular motors that metabolically act as force generators (such as actin/myosin interactions in muscles and cells, actin polymerization in filopodia, microtubule-based systems in cell division as well as flagella and cilia) tend to be comparably slow (e.g., [75, 76]).^10^ However, numerous active elements collectively working together can make things relatively faster (e.g., [77, 78]), affecting important physiological functions such as cardiac muscle contraction [79]. One illustrative example are the flight muscles, which can oscillate at frequencies of 20–1000 Hz (e.g., hummingbirds clock in at 50–80 Hz, while bumblebees at 250 Hz; small midges are the fastest) [80, 81]. Another biomechanical example is the voluntary action of an “ultrafast” fingersnap, which takes several milliseconds [82].

However, the ear is faster in that it encodes mechanical oscillations at frequencies upwards of 1–100 kHz, and perhaps even higher for some cetaceans. At least for mammals, this process occurs in part because of a motor protein called prestin, which has been argued to be unique in that it “is a direct voltage-to-force converter” [83], similar to a piezo-electric material. Further, the ear can encode signals on a cycle-by-cycle basis up to several kHz [84]. This aspect, commonly referred to as “phase locking,” factors prominently into discussions on functionally important topics such as sound localization and temporal versus place coding. Some animals, such as the barn owl, can push this limit even higher, upwards of 10 kHz [85]. From this viewpoint, the ear is several orders of magnitude faster than the eye.^11^

To expand, what makes the ear remarkable in the context of speed is how it pushes past the limits of biophysical “speed limits” such that a hair cell can encode precise timing information to an auditory nerve fiber. Two specific considerations bear consideration. First, the cell membrane itself acts like a low-pass filter by virtue of its resistive-capacitive (RC) time constant [55]. Second, exocytosis of neurotransmitter across the synaptic cleft between the hair cell and neuron also can only occur so fast [87]. Both these factors, and likely others, impact how timing information gets lost above a few kHz and lead to a falloff in phase locking. The structure of the synapse appears relatively optimized to not only be fast (i.e., timescales of 1 ms, [88]), but also capable of maintaining sustained responses leading to functional aspects such as “our ability to detect submillisecond differences in sound arrival at our ears” [89]. Further, the cochlea appears to have found means to still allow for cycle-by-cycle amplification well above 10 kHz (e.g., [90]), even if neural phase locking cannot maintain such speed. Historically, the RC time constant was considered a barrier to high frequency amplification, however, some more recent models posit mechanisms that could circumvent this barrier by trading off gain for bandwidth (e.g., [91]). A recent review goes so far to assert “the RC problem is, in practice, a relatively minor physical issue whose importance has been unduly magnified by viewing it through the wrong lens” [92]. Such a consideration appears consistent with the notion that evolutionary pressures have led to optimizations that result from multiple competing functionalities.

### $\rhd$ (G) The ear can detect signals below the thermal noise floor

**Unclear** — This claim benefits from additional consideration because, as a biological detector, near threshold the ear is intrinsically more probabilistic than deterministic. That is, like the retina during scotopic (low-light) vision, a decision as to whether a stimulus is present or not derives from setting a threshold with respect to some underlying probability distribution [93]. This chiefly arises due to the presence of noise, which can manifest in several different fashions in the inner ear. For example, hair bundles experience thermal fluctuations due to fluid motion, and MET channels are subject to stochastic gating (“channel clatter”). Two considerations for how the ear deals with signal detection in noise are as follows. First is temporal integration: Effects of additive white Gaussian noise (for example) can be arbitrarily reduced by increasing the observation time (e.g., [94, 95]). Second, near threshold, the ear is both an active and nonlinear system (as addressed below, and in Claim (K)). Together, these considerations give rise to provocative notions such as “that Brownian motion of the hair bundle provides an optimal noise level that enhances the sensitivity of MET to weak signals” [96]. Subsequent studies have extended these sorts of considerations (e.g., [97–99]), but broad consensus is still not present. As such, current evidence has not sufficiently confirmed such a notion, and as a result this is a challenging claim to properly address.

Perhaps the best-known argument was provided by Bialek [4, 93], who posited that the threshold of response is about an order of magnitude lower than the noise thermal noise floor. Let us hash out the basics of this argument, which involves some key concepts from physics. However, reader beware — prior remarkable claims were made by Bialek about the ear being quantum-limited (i.e., “the sensitivity of the ear reaches a limit imposed by the uncertainty principle”) [100, 101], which ultimately turned up erroneous [93, 102].

As the primary MET transducers, Bialek’s discussion focused on hair cell bundle deflections. More specifically, his argument [4, 93] is chiefly energy-based and framed around how much a hair cell bundle moves due to a threshold-level stimulus relative to the thermal noise that it is subject to. Heuristically, he considers the bundle as simply free-standing (i.e., ignore overlying tectorial structures) and basally embedded in a simple epithelium that exerts no relevant inter-cellular forces via their somatic bodies. Considering only passive hair cells, he ignored nonlinear active forces that would inject energy into the response: bundle movement solely derives from the external forces acting upon it. The bundle was modeled as a second-order system that has both a stiffness (e.g., due to pivoting at the stereovilli rootlets) and an inertia (e.g., entrained fluid about the bundle). Thus, the bundle is treated as a damped, driven harmonic oscillator driven by noise.

With these assumptions in place, the fluctuation-dissipation theorem (FDT) can be applied to predict how much a bundle moves in response to noise ([4]; see also [103]). This theorem is often described by virtue of the *Langevin equation* [104], a stochastic differential equation (i.e., a system driven by a noise term), and represents an important application of linear systems theory. In brief, the FDT indicates a balance between damping (e.g., fluid viscosity), which causes a system to move towards zero motion, and noise (e.g., thermal bombardment by fluid molecules) that keeps the bundle in motion [104]. In the simplest case, the fluid itself is both the source of the damping and the noisy drive. Statistically speaking, when the system is at thermal equilibrium (i.e., *steady-state*) there is a balance between thermal and free energies, such that the system’s behavior is well-described by average quantities such as the root-mean-square (RMS) displacement.^12^

This modeling approach then requires an upper bound on bundle stiffness, which Bialek took to be $\kappa \sim 10^{-3}$ N/m. A similar model was employed by [105] to estimate bundle stiffness, which was confirmed using glass probes of known stiffness. With these assumptions in place, the “Brownian motion of the bundle” would have an amplitude of $x_{\mathrm{{RMS}}} = \sqrt{k_B T/\kappa } \sim 2$ nm. A different modeling approach for the mammalian cochlea [99] reported a similar value range of 1.2–2.7 nm. Ultimately the degree of damping matters too, a facet we will return to shortly.

How much does thermal noise affect an actual hair bundle? Experimental evidence for bullfrog saccular hair cells decoupled from an otoconial mass [96, 105] and turtle auditory hair cells with the overlying tectorium removed [106] indicate a RMS amplitude of approximately 3–4 nm. This value is for a passive noise-driven bundle, not one undergoing spontaneous oscillations stemming from active processes (see Claim (K)). Further, experiments with bullfrog saccular hair cells suggests that removal of the overlying load (i.e., otoconia) can have significant effects [107], further supported by theoretical analysis [108].

**Fig. 4:**
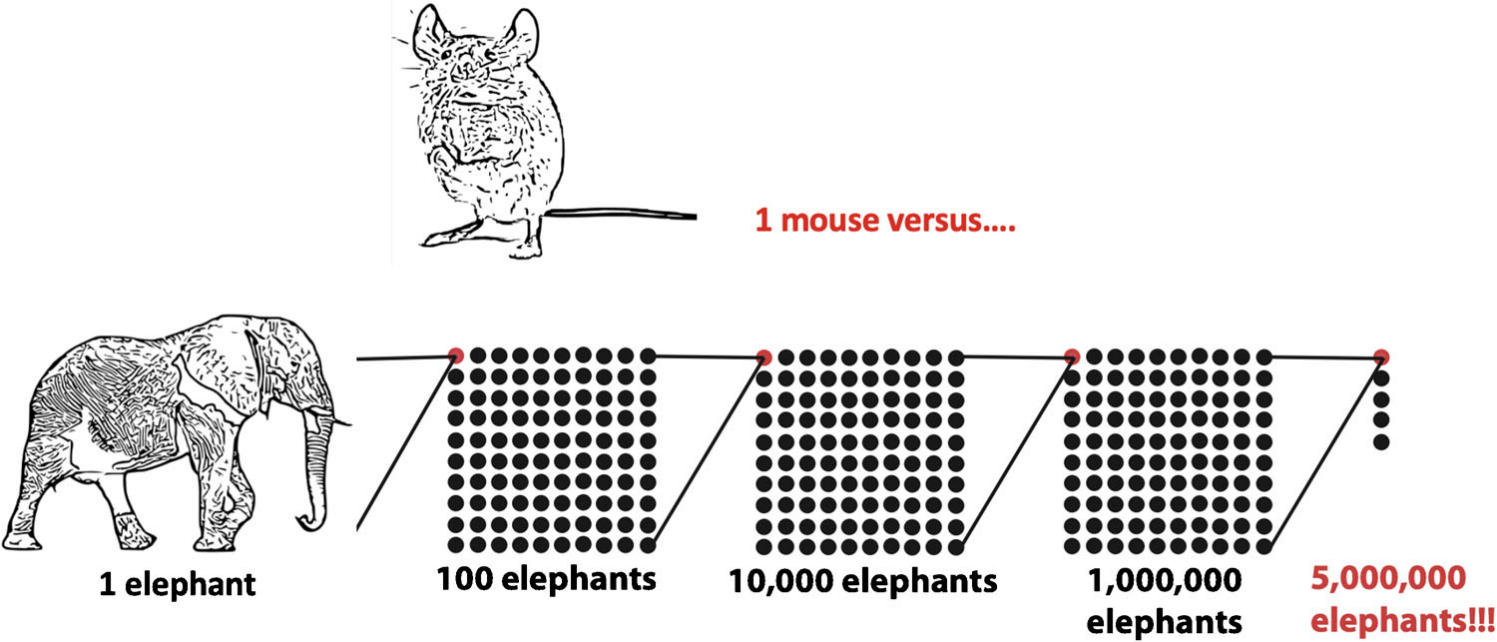
To illustrate the range in sound energy between the threshold and the upper limit where irreversible hearing damage occurs, this figure shows an analogous energy range: the gravitational potential energy of one mouse compared to that of five million elephants

By extension, how much do external sounds at threshold levels move the bundle? As noted earlier, the sound-induced motion of the eardrum at the threshold is on the order of a picometer (i.e., $10^{-12}$ m). What about the resulting movement of the basilar membrane? Various sources suggest BM motion at threshold is on the order of 100 pm (i.e., $10^{-10}$ m, or 0.1 nm) [12, 90, 109, 110]^13^.

These calculations appear to support the argument that thermal noise agitates hair bundles roughly an order of magnitude more than external sound close to the threshold. However, there are several issues with this line of argument. First, the noise-driven amplitude of bundle motion for the mammalian cochlea in vivo is not known. Second, the ear is active in that metabolic energy is used to somehow improve performance. Lastly, and perhaps most importantly, signal detection at threshold is not done by a single hair cell. The ear is a complex system comprised of many cells working together cooperatively [116]. Arguments based upon the movement of a single bundle may be misleading since detection requires spatial and temporal integration.

To conclude, we revisit an analogy relating hair bundle motion to that of a skyscraper [109]. Dallos notes that at the threshold, the basilar membrane (BM) moves on the order of 100 pm. From there, the (external sound-induced) bundle displacement at the threshold can be scaled relative to a movement of several centimeters at the top of the Sears Tower — a small displacement indeed. To extend the analogy, one can consider wind-induced sway in buildings. Too much sway and building occupants can sense it (e.g., leading to vertigo), and too little means the building structure is too stiff. One engineering approach is to build tall buildings as “smart structures” [117] that use some sort of feedback control (e.g., the “tuned mass damper,” [118]), which can be passive or active (e.g., [119]). So perhaps Bialek’s arguments summarized above cannot definitively address this section’s claim, but they could be reframed as suggesting that hair cell(s) act as (biological) smart structures [120].

### $\rhd$ (H) The ear accommodates an enormous dynamic range in terms of incident sound energy $\rhd$ (I) The input range of the central nervous system (CNS) is much smaller than that of audible sound levels

**True & True** — Of course, “enormous” and “much smaller” are relative terms and need to be properly recast into meaningful qualifiers to support/refute these claims. The term dynamic range refers to the span from sound pressure at the threshold (“softest”) to sound pressure levels at which damage readily occurs (“loudest”). In general, the dynamic range of hearing spans 6+ orders of magnitude (i.e., a million times) in terms of sound pressure (i.e., 0–120 dB SPL). As intensity is proportional to the square of pressure, that amounts to 12+ orders of magnitude of energy (e.g., [121]). Correcting an analogy made by Dallos relating the weight of a mouse to an appropriate number of elephants, Fig. 4 shows a span of 12 orders of magnitude in terms of gravitational potential energy (which is proportional to mass): a mouse compared to that of five million elephants. Another back-of-the-envelope analogous range: the energy contained in a D alkaline battery ($10^{5}$ J) to that released in the volcanic eruption at Tonga in 2022 ($10^{17}$ J) [122], which according to the BBC was “the largest atmospheric explosion recorded by modern instrumentation.” From this perspective, “enormous” is a reasonable qualifier. Comparatively speaking, vision appears to have a smaller dynamic range of 8.5 orders of magnitude, considering the (energy-based) illuminance from the dark nighttime and bright daytime skies [123].^14^

**Fig. 5:**
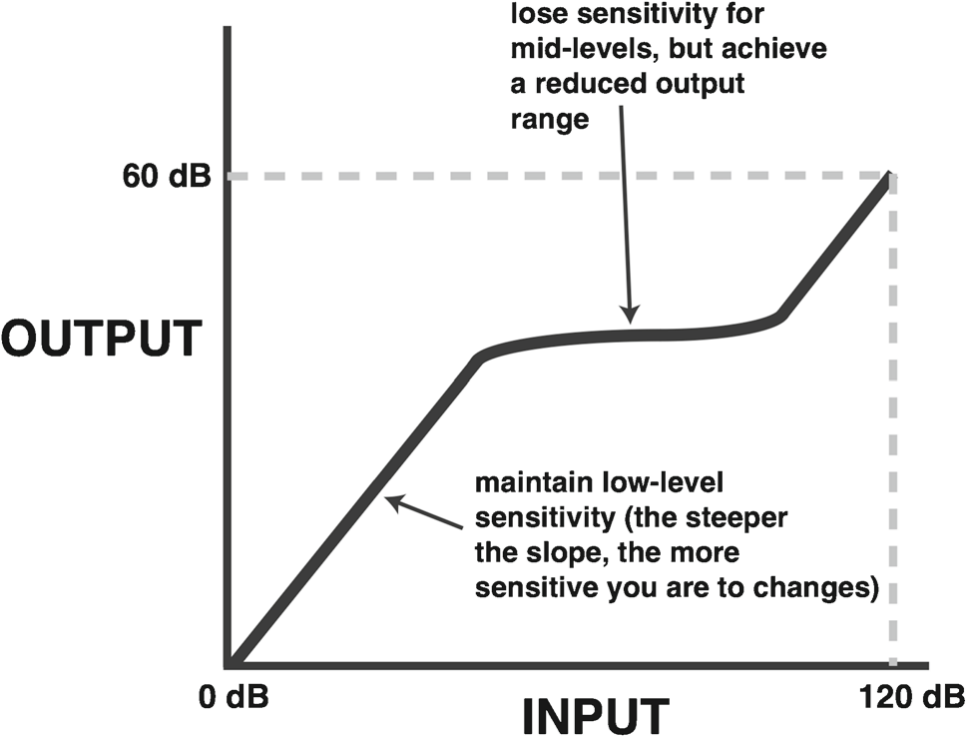
Simple schematic to illustrate various aspects of compression, which allows for a larger dynamic input range to be encoded into a smaller output one. Here, a constant gain at lower levels leads to a linear response, while the decreasing effect of the gain at moderate levels leads to the compressive region. At the highest levels, the effective gain is unity (and hence a linear response again). See also [128]

At any given instant, however, the ear is not readily capable of operating over such a wide range. For example, both the middle ear muscle reflex and efferent modulation from the central auditory system regulate cochlear function. As a result, a key functional aspect of the inner ear is that it behaves in a nonlinear fashion to provide “compression” [125, 126], as shown in Fig. 5. That is, the aforementioned large dynamic range (100+ dB) of inputs is compressed into the smaller output range of the auditory nerve and central nervous system.

The nervous system may further limit the dynamic range of hearing. Single-unit auditory nerve fibers have a fairly limited range of variable firing rates between spontaneous firing and saturation, which thereby affects encoding to the central nervous system. Consider this quote from [127]: *“The afferent nerve similarly has a restriction on its dynamic range. The maximum sustainable rate of action potentials is on the order of a few hundred to a thousand per second while a reasonable minimum would be on the order of ten or so per second. Rates slower then this could not pass information quickly enough. Hence, the dynamic range of an afferent nerve fiber would be around 10–100:1, or between 20 and 40 dB.*” Another source [34] suggests even lower spontaneous firing rates (0.5 spikes/s), leading to a dynamic range of 20–60 dB.

Given that each inner hair cell is innervated by numerous auditory nerve fibers, and those neurons can have a variety of spontaneous firing rates (e.g., due to different synaptic coupling strength), it appears that a variety of variable spontaneous rate auditory nerve fibers is sufficient to encode a large range [34]. However, hair cells themselves also exhibit a limited range, in part due to the sigmoidal nature of the MET channel [129]. We therefore need to consider the dynamic range of a hair cell (and thereby “the ear”) within the context of an active ear, as described in the next pair of claims.

### $\rhd$ (J) The ear not only detects sound but emits it as well $\rhd$ (K) The ear has a built-in amplifier

**True & True** — The (healthy) ear not only detects sounds but also generates and (coherently) emits it as well [130–132]. These sounds called otoacoustic emissions (OAEs), are readily measurable and have had profound implications for scientific research and clinical applications since their discovery in 1978. A detailed historical account of their discovery and uses has been described [133]. It is well-established that these emissions are a fairly universal feature across the animal kingdom [7, 134–136], despite vast differences in auditory morphology.

The existence of otoacoustic emissions is commonly taken as evidence for *a*ctive energy-producing processes in the ear. This line of reasoning is perhaps most compelling for spontaneous otoacoustic emissions (SOAEs) since they can exist in the absence of external acoustic stimulation. Furthermore, statistical properties of SOAEs are consistent with self-sustained oscillations, as opposed to filtered noise [100, 137], which has been interpreted as evidence for metabolically-based force generation that can lead to power amplification [138]. OAEs are then (presumably) a by-product of such processes.^15^ Properties of SOAEs have also been shown to correlate with perceptual measures of hearing such as threshold minima [140, 141] and frequency difference limens (e.g., [142]). In combination with the previous argument that SOAEs result from active, energy-producing processes, the correlation of OAEs with perception suggests a direct linkage of perceptually important phenomena to active processes. For additional considerations along these lines linking OAE activity and active processes, see [143].

There is a wealth of evidence demonstrating OAEs in multiple taxa, suggesting that the mechanisms responsible for otoacoustic emission generation are adaptive for hearing. Despite this evidence, however, direct empirical validation of active force generation in the ear has been challenging and controversial. As such, there are theories that challenge the notion of an active ear (e.g., [144, 145]). Why is it so hard to measure active force generation in the mammalian cochlea? Aside from the coiled nature of the cochlea, consider other sections of this review, including the hardness of the temporal bone (Claim (B)) and the fragility of inner ear structures (Claim (P)). While von Bekesy’s remarkable contributions used excised postmortem tissue [146], it was not until much later that the knowledge of nonlinearity started to emerge by studying living ears (e.g., [147, 148]). Work with nonmammals will certainly help clarify amplification manifests, though the debate will linger on the applicability of such to mammals. Is the ear active? Yes, but a definitive measure of cochlear amplification is still at large.

### $\rhd$ (L) The ear has a unique “fingerprint”

**Fig. 6:**
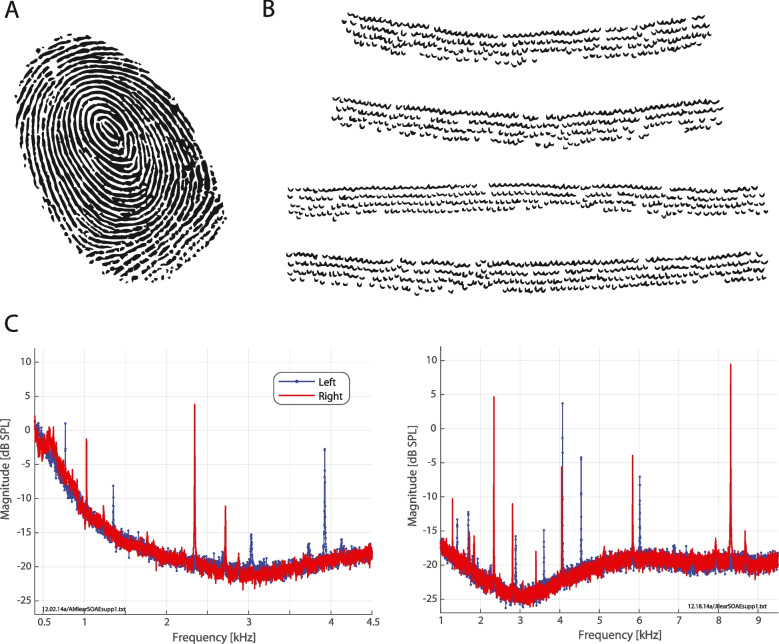
**A** Schematic of a typical fingerprint (obtained from the Wikimedia Commons, where it is in the public domain). **B** Example of a cochlear “fingerprint,” apparent as a “roughness” in hair cell arrangement. Adapted from [158], this shows OHC layout in several different contiguous cochlear sections from a non-human primate (Rhesus macaque, *Mucaca mulutta*). These sections are roughly 1/3 of the cochlear length from the apex and represent about 10% total of the total length. Each small v-shape represents a single OHC bundle. While there is a crystalline-like structure (e.g., clear rows of hair cells), there are clearly also irregularities (e.g., buckling of a row, rotated bundle orientation, extra hair cell packed closely in). **C** Functional examples of cochlear fingerprints as arising otoacoustically. These SOAE spectra (from two different normal-hearing human subjects, in the left and right panels respectively) indicate the unique spectral peaks that manifest in the ear canal. Furthermore, spectra from both left and right ears are shown for a given subject, indicating each cochlea yields somewhat unique SOAE patterns. Remarkably, these two subjects both exhibited a large SOAE peak in their right ear right around 2.34 kHz

**True** — The outer ear (i.e., pinna shape) has been proposed as a useful biometric marker [149], similar to facial recognition. Perhaps more interesting is the consideration that the inner ear may have a unique functional “fingerprint”. That is, there is a spectral fingerprint unique to a given ear where certain frequencies are encoded/perceived differently than others [150]. One commercial application is the “personalized listening” provided by headphones produced by the company Nura (and acquired by Denon), which measures a person’s sound-evoked OAEs as a pre-filter for sound presentation through the headphones. Relatedly, spectral magnitudes of OAEs from a given ear have a unique set of peaks and valleys that are closely related to threshold microstructure [140, 150, 151] and unique patterns of SOAE activity (e.g., Fig. 6C). What causes this spectral fingerprint?

Many models of inner ear function make explicit assumptions about “roughness” (e.g., [152–155]). The basic idea is that there is some sort of variation across individuals but constant for a given ear, that serves as a biomechanical foundation to this fingerprint (Fig. 6A). One possible mechanism could be variation in hair cell location along the cochlea (e.g., Fig. 6B shows an example for a rhesus monkey). However, precisely what morphologically and/or physiologically constitutes roughness is an open question and important in future hearing research (i.e., to empirically confirm a key theoretical assumption). Another facet worth mentioning is that in some non-mammals such as lizards, interaural acoustic coupling can allow for the two ears to effectively synchronize [156, 157]. Thus, in some instances, a “fingerprint” may not be unique to just a given ear, but the entire auditory periphery.

### $\rhd$ (M) Cochlear waves play a crucial role in hearing

**True** — Since the discovery of cochlear traveling waves by Bekesy, who won the 1961 Nobel Prize in Physiology or Medicine for his work on hearing [146], there has been intense and protracted debate about precisely what role these waves play in auditory transduction. For brevity, we will not rehash these arguments but instead point interested readers towards several relevant papers: [110, 159–161]. Bekesy himself was well aware of these considerations and strove to find an appropriate heuristic for the role of waves in the ear^16^. Ultimately, the physiological data are unequivocal: a variety of wave-like behaviors are at work in the mammalian cochlea (e.g., [162]). Further, such is likely to be present in other ears where basilar membrane traveling waves appear absent, such as lizards and frogs (e.g., [163–165]) demonstrating that these waves are likely adaptive for hearing across taxa. While further study is needed, presumably, wave behavior in the inner ear implicitly leads to relative phase relationships between key constituent components that affect energy flow and thereby how elements cooperatively work together [116, 166]. For example, one broadly accepted theory of cochlear mechanics considers “coherent scattering” [152] as a key biomechanical principle, where collective force generation can effectively be focused to functional benefit. Such a consideration has broad comparative and evolutionary implications [7, 167], as recently explored in two comparative studies examining SOAE generation [168, 169] and further expanded upon in the next claim.

### $\rhd$ (N) The ear is poised on the verge of an instability

**Unclear** — On one hand, the presence of SOAE activity suggests this claim is true, as if SOAE might be akin to acoustic feedback in a microphone/speaker setup (as experienced during the “sound check” at many music venues!). On the other hand, however, this is a challenging claim to address given that the inner ear is a nonlinear, noisy, non-equilibrium system and thus the appropriate point of reference is unclear: how do we first define stability? Consider a simple (1-D) mechanical analogy: a ball on a landscape of hills subject to a uniform downward gravitational force as well as friction. If the ball is down in a “valley,” the rest point (i.e., if you put the ball there, it stays there; also called an equilibrium point) is stable. That is, a small perturbation will not cause the ball to move significantly away, and eventually the ball would come back to the rest point. However, if you balance the ball just right at the top of a hill, that rest point would be unstable because a small perturbation would cause the ball to roll downhill (i.e., away from the rest point). In the language of linear differential equations (e.g., harmonic oscillator), the real part of an eigenvalue associated with an equilibrium point is negative for a stable point and positive for an unstable one.

Now consider an active nonlinear oscillator such as the van der Pol system. The van der Pol equation is a useful heuristic, as it has been used to great benefit for early studies of SOAE generation (e.g., [170]). The associated equation of motion for the undriven case (i.e., autonomous) is $\ddot{x} - \mu (1-x^2)\dot{x} + x = 0$ with “control parameter” $\mu$. As $\mu$ goes from zero to a positive value, the system undergoes a supercritical Hopf bifurcation that causes it to behave as a “limit-cycle” oscillator (i.e., it self-oscillates due to the nonlinear damping for small displacements, which is stabilized by the cubic nonlinear term for larger displacements). Is such considered unstable? On one hand, this behavior is not stable in the traditional sense as there is no stable equilibrium position ($x=\dot{x}=0$ has positive real parts to the eigenvalues upon linearization). On the other hand, however, the limit-cycle behavior is a reliable and predictable feature: perturbations would eventually “relax” back to the self-sustained oscillation (hence why this sort of system is sometimes referred to as a “relaxation oscillator,” a term coined by Balthasar van der Pol [171]).

While the inner ear is not simply a limit-cycle oscillator, it has been suggested that there are two fundamentally different camps for considering how amplification arises in the inner ear and these arguments consider the “instability” of the system [116, 172]. On one end of the spectrum, experiment, and modeling with bullfrog saccular hair cells have shown that (in vitro) they can behave like limit-cycle oscillators [173, 174]. By extension, the argument becomes that the inner ear can be considered as “an array of critical oscillators”, with hair cells poised at a “Hopf bifurcation” [174]. From this perspective, the ear is indeed poised near instability. On the other end, the argument is that the ear is considered a distributed system of disparate parts working together and ultimately “the cochlea is poised elsewhere” [116] (see also [137, 175]). That is, the ear is intrinsically stable because active energy created in one location can readily be dissipated elsewhere. In summary, the distinction here perhaps boils down to Reductionist versus Gestalt viewpoints about how the inner ear works, as motivated in the preface to [167] (see also [169]). Regardless, as indicated with respect to Claim (G), empirical evidence is scant in terms of what is in fact oscillating spontaneously inside the inner ear to generate SOAE, as well as the phasic relationship between various moving parts. Without such evidence, arguments associated with this claim are likely to persist.

### $\rhd$ (O) The ear is highly nonlinear

**False** — In order to best preserve information from external acoustic signals, one might expect the ear to exhibit relatively high “fidelity.” At face value, this would be best achieved by transduction ultimately being as linear as possible. Nonetheless, several physiological and functional considerations indicate nonlinear effects play a prominent role in hearing [176]. At a crucial stage in the transduction process, hair cell MET is intrinsically nonlinear given the sigmoidal relationship between bundle displacement and transduction current [177]. Other physiological aspects of hair cells also exhibit nonlinear behavior, such as nonlinear OHC capacitance [177]. These nonlinearities have salient effects. For example, the distortion of sound is easily, albeit faintly, perceived (e.g., Tartini tones, sometimes referred to as “auditory illusions”). Further, consider the ubiquity of distortion product emissions (DPOAE) as a pediatric audiological tool: A linear ear would not produce such emissions. That is, it is normal for a healthy ear to produce intermodulation distortion in the ear canal in response to sound (see also Claim (J)). There are clear hallmarks for nonlinear phenomena in hearing.

The interplay between the linear and nonlinear functional aspects of hearing further manifests with the common notion that the inner ear acts as a hydrodynamical Fourier analyzer, where the frequency is mapped to space (i.e., tonotopy). Taking into account distortion products, compressive behavior, and two-tone suppression, Mather (2016) asserts: “Such gross departures from linearity clearly rule out the view that the ear performs a strict Fourier analysis of the incoming sound signal. However (...) the ear can be viewed as an approximation to Fourier analysis.” [34]. So it appears Mather is taking the middle ground here: the ear is nonlinear but achieves a functionality consistent with a linear system.

It is thereby with regard to “highly nonlinear” that the argument becomes more nuanced. On one hand, nonlinear aspects could arise from a variety of causes but produce desirable characteristics such as compression (Fig. 5; see also Claim (H)). But on the other hand, there could be clear drawbacks to a high degree of non-linearity, such as chaotic behavior. Chaos is a well-defined concept in dynamical systems theory, and can readily arise in simple nonlinear systems [178]. While there are numerous examples of chaotic behavior in biological systems (e.g., [179]), challenges exist in assessing whether observed data are ultimately indicative of a chaotic system [180]. Chaos is most commonly associated with a sensitivity to initial conditions that can allow for deterministic systems to appear noisy. Indeed, nonlinear models of the inner ear can exhibit a relatively high degree of such sensitivity (e.g., [154, 168]). Several studies have argued for the existence of chaotic behavior in the ear, both in terms of OAE [181] and hair cell bundle motions [182], and that chaos can even be beneficial [183]. However, features of the active ear such as SOAEs are generally quite stable over large timescales (e.g., decades, [184]). A clear picture has yet to emerge as to whether chaotic dynamics play any meaningful role in auditory transduction.

In summary, we argue that to best encode sound, the ear should be both as linear as possible (to maintain fidelity) and nonlinear to an extent that allows for desirable features to emerge (e.g., compression to increase the dynamic range). Given this interplay, we further argue that too many facets of auditory function behave in a linear fashion to conclude that the ear is “highly” nonlinear. We add the following statement from [185] as a corollary: “It is somewhat surprising that linear analysis can account for so many features of the cochlea when it is inherently nonlinear.”

### $\rhd$ (P) The ear is very fragile

**Both True & False** — As noted earlier, the (mammalian) inner ear appears to be fully encased in one of the hardest bones in the body. Such may not only allow for receptivity to signals via bone conduction but may also afford both physical protection by providing insulation from the outside world. Unfortunately, this encasement also makes intracochlear physiology exceedingly difficult.^17^ Regardless, the prevalence of hearing loss (HL) indicates that the ear is clearly susceptible to damage.

There are several underlying causes of acquired HL. The most common, age-related HL (also called presbycusis), is described as a “multifactorial process” with likely contributions from vascular and sensorineural dysfunction [186]. Exposure to high-level sounds can cause both temporary and permanent types of hearing loss (noise-induced hearing loss, or NIHL), and NIHL is likely a compounding factor in presbycusis. The inner ear is also vulnerable to various pharmaceutical and chemical agents such as aminoglycoside antibiotics and the chemotherapy agent cisplatin (see [187] for a review). Each of these causes (age, noise, ototoxin exposure) is associated with hair cell loss, but recent work also suggests a correlation with cochlear synaptopathy, sometimes referred to as “hidden hearing loss” (e.g., [188]), a form of sensorineural hearing loss that is currently undergoing extensive research and modeling [189]. Lastly, HL can manifest more centrally (e.g., cortical level) due to various types of noise exposure [190] and give rise to conditions such as tinnitus that are challenging to address clinically. These examples illustrate cochlear fragility to a variety of intrinsic and extrinsic factors.^18^

Additionally, some ears may be more resilient than others. Differences in efferent innervation (i.e., neural “wiring” to the cochlea) [193] provide a two-way street between the ear and brain that may affect the ear’s susceptibility to damage. Thereby, the ears of some individuals may be less “fragile” than others. What factors determine relative “toughness”? It appears there is a fine line between the degree of noise exposure that may be beneficial towards improving olivocochlear effects versus causing hearing loss (peripheral and/or central) [190]. Regardless, the claim of “fragility” is both supported and expanded by this context.

Nonetheless, it can also be argued that the ear is not fragile at all. That is, despite its complexity and metabolic demands (e.g., see Claim (E)), it generally maintains its overall functionality for the entire lifespan with only minor degradation. Seen from this point of view, age-related HL is relatively minimal given all that an ear is typically exposed to on any given day, day-after-day, for decades. And in the case of non-mammals, hair cell regeneration leads to restoration of functional hearing after various types of damage that would leave a mammal profoundly deaf [194–196]. While the inner ear is susceptible to damage and therefore could be considered fragile, viewed in light of long lifespans and daily metabolic demands, perhaps the ear is more robust than is widely believed.

### $\rhd$ (Q) Human hearing exhibits relatively exceptional tuning sharpness

**Partially True** — In a basic sense, the ear has two functional characteristics that allow a listener to make auditory discriminations: sensitivity and selectivity. Sensitivity at threshold refers to the ability to be able to detect the presence of a sound in quiet, whereas (frequency) selectivity relates to distinguishing two (or more) different sounds based upon their spectral content.^19^ Frequency selectivity is often used synonymously with “tuning”: more selective equates to sharper tuning. The ear achieves this latter aspect of tuning by virtue of mechanical tonotopic mapping: Different frequencies correspond to different spatial locations along the length of the cochlea (or inner ear).

A long-standing assumption in auditory neuroscience was that humans exhibited frequency selectivity similar to other mammals, as reviewed in [197]. However, it was proposed that by employing otoacoustic and revised psychoacoustic methods using forward-masking, frequency selectivity in humans is in fact relatively sharper [198, 199]. This notion proved controversial (e.g., [200, 201]), but additional studies subsequently solidified the notion that OAE-based tuning estimates correlated well to neurophysiological ones [197, 202, 203]. It is thus now reasonably well-established that humans do in fact exhibit sharper tuning than other mammals that are commonly used for auditory neuroscience.

There is a degree of subjectivity with regard to “exceptional,” however, in that there are likely other less-studied animals that exhibit relatively sharper tuning over a broad frequency range. Consider for example larger mammals, such as elephants and cetaceans, that have rcomparably long cochleae [25, 49]. While one needs to factor in the frequency range of hearing to determine a space constant (e.g., [204]), it may be the case that these larger ears have more tonotopic “real estate.” One OAE-based study comparing tuning estimates between humans and tigers, the latter having a longer cochlea [205], found that tuning estimates were comparable between the two [206]. Determining frequency selectivity in cetaceans is challenging for a variety of reasons, but estimates indicate relatively sharp tuning [207, 208] and further study would be required to properly compare these values to those of humans.^20^ So in short, evidence indicates that human tuning is sharper compared to some, but not that there is reason to believe it is exceptional. Further, tuning that is too sharp comes at the expense of temporal acuity. This stems from filter theory predicting an inherent trade-off between frequency selectivity and temporal resolution (e.g., [202, 213]). That is, sharper tuning inevitably leads to poorer time resolution, so greater frequency selectivity comes at a cost. Thus, presumably a given species strikes a balance to optimize both morphological and ecological facets unique to their situation. What precise factors led to the present balance for humans remains open to debate.

## Summary

In this paper, we examined several claims regarding “remarkable” properties of the ear. While we recognize these claims are inherently subjective, their detailed examination provides fertile ground for many key topics in auditory science. For example, consider how many of these claims are intrinsically linked, such as how a “built-in amplifier” (K) leads to “detect(ion of) signals below the thermal noise floor” (G) and an “enormous dynamic range” (H). Further, consider hearing through an evolutionary lens [49, 167]. In a broader comparative context, how many of the stated claims explored here change when examining the multitude of morphological and functional variations across the ears of creatures inhabiting this planet? The associated evolutionary pressures have led to a myriad of biomechanical solutions to “hearing,” much in a similar yet distinctly different fashion when compared to vision [124]. Future research will certainly provide greater insight on how truly remarkable the processes are that allow us to hear the world around us.

## Data Availability

N/A

## Footnotes

1 This value for threshold displacement is inferred by extrapolating the displacement of approximately $8\,$pm measured with a sound pressure of approximately $200\,\mu$N/m$^2$ to a sound pressure near the threshold of hearing (approximately $20\,\mu$N/m$^2$) and rounding up to $1\,$pm. What is less clear is whether this number is larger or smaller than the thermally induced motions of the eardrum that result in the absence of any stimulus. Other plots from [3] suggest it is comparable, though also indicate that caution is needed in extending their Fig. 1 estimates down to 0 dB SPL.

2 Another example for the small end of the spatial scale of hearing (i.e., 1 pm): “*In a sense, a picometer is kind of the ‘scale of antimatter’. When an electron and positron annihilate, they make two 2.4 picometer wavelength photons. Alternatively, if one were to consider 1/pm as an energy, it is around the minimum energy you need to make the lightest antiparticle (i.e., a positron)*.” (S. Tulin; unpublished) Here, “1/pm” refers to the energy of an incident photon (via the Plank relation) required to create a positron in pair production, which in this case would mean a gamma ray.

3 By linearity, we specifically mean a linear system that exhibits the key properties of additivity (i.e., superposition) and homogeneity (i.e., proportionality between input and output). Further, a system that does not possess both these properties would be considered nonlinear. Nonlinear systems can exhibit a wide variety of behavior, ranging from linear-like to more “complex” phenomena such as chaos. See Claim (O) for further expansion in this regard.

4 The hardness of something, though empirically quantifiable, stems from other material properties such as Young’s modulus and density [20]. Furthermore, comparative studies of bone (e.g., [21]) note that mechanical properties like “hardness” are not only tied to biochemical aspects such as the degree of mineralization, but functional aspects as well. Direct approaches to measuring hardness may include “work of fracture, bending strength, and modulus of elasticity in bending” [21], using the force required to produce a “crack” as a reference point. While indirect (i.e., radiometric) temporal bone density has been reported in numerous clinical contexts, these density values usually serve as proxy measures for other considerations such as otosclerosis progression (e.g., [22]) or surgical planning (e.g., [23]). Thus, we are not aware of absolute measures of temporal bone density, nor of a systematic comparison in bone density measures across the body.

5 Older texts describing the morphology of the vertebrate skeleton sometimes indicate that there are four ossicles: the commonly mentioned three as well as the “lenticular” which was situated between the incus and stapes. This latter fourth ossicle was referred to as “smaller than the others and is sometimes not distinct” [38]. At face value, such would likely constitute the smallest bone. However, it appears that most anatomists have come to the conclusion that the lenticular is in fact the “lenticular process” of the incus and not separate in of itself [39]. Nonetheless, some controversy has continued, with assertions such as “the lenticular process is the smallest bone in the human anatomy” [39, 40]

6 On the opposite end of the spectrum, the femur (or “thigh bone”) is commonly pointed to as both the largest and “strongest” bone in the body, although precisely what the latter qualification means can be poorly defined. A rough estimate for the volume of the femur yields approximately $3\times 10^5$ mm$^3$. Considering the stapes as a thin disk, the dimensions listed above indicate a volume of about 10 mm$^3$. Thus, to a rough first order, one might expect something on the order of ten thousand copies of the smallest bone in the human body to fit within the largest bone.

7 It is worth noting that the middle ear ossicles play a significant role in bridging current theories of evolution with archeological record (e.g., [50, 51]). There is controversy over how the ossicles evolved, as well as how the relative size of the stapes has decreased. As such, there have been many comparative studies of the middle ear across mammals (e.g., [52]), albeit fewer for non-mammals where major differences arise.

8 Here we briefly define resting potential. A key function of cell membranes is simply to separate inside (i.e., intracellular) from outside (extracellular), in particular, ions such as sodium (Na+), potassium (K+), and calcium (Ca++). As such, an electrical potential can be set up on either side of the membrane due to charge separation and selective permeability. The resulting potential is easily described as a weighted sum of Nernst potentials via the Goldman-Hodgkin-Katz equation. Changes in the permeability to the membrane can affect the instantaneous potential at any given time (e.g., generation of an action potential). But in its normal steady-state conditions, the potential is referred to as the resting potential [55] and effectively serves as a battery to help fuel cellular transport processes.

9 Typically, intracellular fluids have a low Na+ concentration and a high K+ concentration. Conversely, extracellular fluids have a relatively high Na+ concentration and a low K+ concentration. What makes the cochlea unique is that there is a high extracellular K+ concentration, making it a dominant ion flowing into the cell via the MET channels.

10 Molecular motor speed depends upon several factors, such as external ATP concentration and the load. Using higher estimates from [75], a single kinesin motor protein would take anywhere from 0.3 to 3.5 days to march across a dime (about 18 mm in diameter)!

11 Given the association to hearing, vocal production can also be considered fast, though some animals such as the blue-throated hummingbird create sounds that are too high in frequency for them to actually be able to hear [86]. However, vertebrate vocal production generally does not appear to involve cycle-by-cycle force generation, but instead the passive vibration of structures (e.g., larynx, syrinx) driven by broadband phonation.

12 Thermal-driven motion is noisy, like picking a value from a Gaussian distribution. An RMS value thus provides a reasonable quantitative measure of how much motion one might expect on average (independent of direction).

13 Other reviews have reported similar values (e.g., [4]), suggesting even “subangstrom” values for the cat down to 5 pm for BM responses inferred at threshold. Recent data from the mouse using optical coherence tomography (OCT) [111] indicates BM motions of approximately of 5–10 nm at 20 dB SPL for a 9 kHz stimulus (presumably the characteristic frequency, CF, for that measurement location). Auditory nerve fiber threshold data for mice [112] suggest thresholds of 5–10 dB SPL at 9 kHz. However, decreasing the stimulus level further at CF causes relatively little change due to highly compressive (i.e., nonlinear; see Fig. 5) level-dependence [111]. Taking 100 pm as the value, that is about approximately 40 dB more than the eardrum. That relative boost appears to stem from both passive mechanics tied to middle ear transmission (i.e., pressure gain of 20–30 dB; human: [113], gerbil: [114]) and via active processes (see next point). How much a hair bundle is stimulated in response to BM displacement is a separate issue, as BM motion is not the only factor leading to hair cell bundle deflection (e.g., relative motion between the reticular lamina and tectorial membrane causes shearing; see [115]). For simplicity, assume bundle deflection near the threshold is similar to the BM and on the order of 0.1 nm. Such is consistent with [109], who noted 0.3 nm.

14 A slightly higher range of $10^{10}$ in luminance is noted by [124]. Also, in a fashion generally similar to the ear, we note that [123] states: “This is wider than our eyes can handle instantaneously or even with long-time adaptation. The sensing and acquisition system in our eyes handles over 5 orders of magnitude in real time and up to 8 orders with long-time adaptation.” As a tangent, the high dynamic range of vision carries over to numerous aspects of machine vision, so much so that the term “High-Dynamic-Range (HDR) Vision” typically is meant to refer to imaging technologies rather than what the retina does.

15 To a rough first order, 0 dB SPL (or $\approx$20 $\mu$Pa) appears to be a universal minimum for threshold sound detection. As discussed for Claim (A), this presumably relates to the jitter of air molecules themselves, as well as some balance between cell-based mechano-detection and active force generation. However, some terrestrial animals (e.g., cat, barn owl) exhibit thresholds to airborne sound below 0 dB SPL at certain frequencies [139]. One explanation might be that those species have found passive means to further boost detection of low-level signals such as acoustic “lensing” (e.g., to “facial disk” composed of the feathers around the barn owls face[139]).

16 Consider for example that Bekesy built a mechanical model consisting of a sequence of pendula with smoothly changing natural frequencies. He showed that little (or no) coupling between sections was sufficient to generate motions that looked like waves. This was important to his understanding of “paradoxical” motion, whereby motions progressed from base to apex even when he intentionally stimulated the apex. Much of this perhaps can be summarized by a pithy excerpt taken from Issac Asimov’s Second Foundation: “*Some had attempted to form classifications of waves into waking and sleeping, calm and excited, well and ill — but even the broadest conceptions had their hordes of vitiating exceptions*.”

17 Consider that in Rhode’s seminal 1971 study, he noted that “nearly 150 animals” were used [148]. This was during a time when non-human primates were more commonly used for research (e.g., one could purchase living monkeys delivered to your home from ads in comic books!). At present, advances in biophotonics are shedding new light on the biomechanics of the inner ear because they can image through bone, allowing for less damage/perturbations to the inner ear.

18 It should be noted that HL is not universally viewed as inherently detrimental. For example, the Deaf community has a broad and rich array of perspectives on how HL need not be considered pathological (e.g., [191]). Further, Deaf people could be considered part of a “linguistic minority group” [192]. So our discussion here is limited to functional considerations, not sociological ones.

19 Without delving into detailed psychoacoustical considerations, we make a simple analogy to distinguish these characteristics by considering two different violin-related “thresholds” scenarios. For sensitivity, one can listen to a string bowed increasingly softly until a sound is no longer detectable. Conversely, for selectivity, one can move the finger holding down a string on the neck (for a given drawing of the bow) ever so slightly until a change in pitch is no longer detectable.

20 Several additional considerations warrant mention. First, is the possibility of an auditory “fovea” in some animals. That is, similar to the foveal vision of the eye (i.e., higher cone density to improve spatial acuity), there may be a localized region of the inner ear that exhibits sharper tuning for a specialized purpose. One example would be for the purposes of echolocation, as demonstrated for bats [209, 210]. Another example is the barn owl [211], which shows an extended frequency range per unit length of the basilar membrane. This may not only affect selectivity, but sensitivity as well. Second, frequency selectivity should not be equated to perceptual features such as pitch discrimination. Selectivity is a peripheral functional consequence of the biomechanics of the cochlea, whereas pitch involves subsequent central neural processing that integrates together responses across time and space/frequency. It is thus important to consider such when examining pitch perception (e.g., [212]).

## Code Availability

N/A
